# Sustainable Development of Magnetic Chitosan Core–Shell Network for the Removal of Organic Dyes from Aqueous Solutions

**DOI:** 10.3390/ma14247701

**Published:** 2021-12-13

**Authors:** Karthik Rathinam, Xinwei Kou, Ralph Hobby, Stefan Panglisch

**Affiliations:** 1 Chair for Mechanical Process Engineering and Water Technology, University of Duisburg-Essen, Lotharstr. 1, 47057 Duisburg, Germany; xinwei.kou@stud.uni-due.de (X.K.); ralph.hobby@uni-due.de (R.H.); 2 IWW Water Centre, Moritzstraße 26, 45476 Mülheim an der Ruhr, Germany; 3 DGMT German Society for Membrane Technology e.V., Universitätsstr. 2, 45141 Essen, Germany; 4 Centre for Water and Environmental Research (ZWU), Universitätsstr. 2, 45141 Essen, Germany

**Keywords:** chitosan, magnetic nanoparticles, dye removal, adsorption, regeneration

## Abstract

The wide use of alizarin red S (ARS), a typical anthraquinone dye, has led to its continued accumulation in the aquatic environment, which causes mutagenic and carcinogenic effects on organisms. Therefore, this study focused on the removal of ARS dye by adsorption onto a magnetic chitosan core–shell network (MCN). The successful synthesis of the MCN was confirmed by ATR-FTIR, SEM, and EDX analysis. The influence of several parameters on the removal of ARS dye by the MCN revealed that the adsorption process reached equilibrium after 60 min, pH played a major role, and electrostatic interactions dominated for the ARS dye removal under acidic conditions. The adsorption data were described well by the Langmuir isotherm and a pseudo-second order kinetic model. In addition to the preferable adsorption of hydrophobic dissolved organic matter (DOM) fractions onto the MCN, the electrostatic repulsive forces between the previously adsorbed DOM onto MCN and ARS dye resulted in lower ARS dye removal. Furthermore, the MCN could easily be regenerated and reused for up to at least five cycles with more than 70% of its original efficiency. Most importantly, the spent MCN was pyrolytically converted into N-doped magnetic carbon and used as an adsorbent for various dyes, thus establishing a waste-free adsorption process.

## 1. Introduction

Dyes and pigments are often used in the textile, paper, food, plastics, and medical industries; hence, they are closely associated with human life [1]. Approximately, 70 million tons of synthetic dyes are produced annually to meet the global demand. Of these, 10% is discharged as wastewater and thus accumulates in the environment, posing a potential threat to the environment [2]. Dyes can be poisonous to the aquatic organisms and cause significant harm to human organs, including the kidney, liver, brain, reproductive organs, and central nervous system [3]. Alizarin red S (ARS) is a typical anthraquinone dye and is extensively used in textile industries as well as in the medical industry as a staining agent; consequently, this has resulted in severe pollution of water with ARS [4]. In addition, it has been found that the ARS is a recalcitrant and durable dye that could induce oxidative damage in organisms and hence is mutagenic and carcinogenic [5]. Thus, it is very important for the environment to remove ARS from contaminated water.

Plenty of methods, including biological methods [6], advanced oxidation processes [7], coagulation and flocculation [8], adsorption [9], membrane [10], and ion exchange [11] are nowadays used to remove dyes from wastewater [12]. Notably, adsorption is undoubtedly one of the most efficient dye removal technologies owing to easy handling, cost-effectiveness, adsorbent flexibility, regeneration ability, etc. Further, it can successfully remove dyes from contaminated water in a short amount of time and results in no secondary contamination to the water body [13]. The adsorbent is a critical component in the adsorption process and has received a great deal of attention as a result. Although activated carbon is commonly recommended as an adsorbent to remove dyes and other toxins, its use is often limited due to its high cost and carbon footprint [14,15].

Nowadays, many research studies have been devoted to the sustainable development of eco-friendly adsorbents for water treatment [16]. In this respect, chitosan, a biopolymer has attracted significant attention owing to its abundance in nature, non-toxicity, and surface functionality [17]. Chitosan and its derivatives play a strong role in the field of water and wastewater treatment due to their polycationic nature and the ubiquitous amino and hydroxyl groups present in their structures [18]. Chitosan-based adsorbents have been shown to have excellent removal capabilities for anionic dyes and heavy metals [19,20]. Nevertheless, common problems encountered in adsorption processes involving very fine chitosan-based adsorbents are the recovery and reuse of the spent adsorbent. To solve this problem, magnetically separable chitosan has been produced and used as an adsorbent in water treatment [21,22,23].

Magnetic chitosan-based materials have been found to be effective in removing several metal ions, such as mercury [24], copper, zinc, lead, cadmium [25,26], and chromium [27]. Besides, various magnetic chitosan-based adsorbents have been used to remove various dyes from water, such as magnetic chitosan/graphene oxide composite [28], magnetic carboxymethyl chitosan aerogel [29], magnetic chitosan/quaternary ammonium salt graphene oxide composite [30], magnetic chitosan nanocomposites modified by graphene oxide and polyethyleneimine [31], pectin/chitosan magnetic sponge [32], alginate beads impregnated with magnetic chitosan@zeolite nanocomposite [33], magnetic chitosan with ARS as imprinted molecules [34], and ethylenediamine-modified magnetic chitosan nanoparticles [35], have been tested for the removal of various dye molecules. Furthermore, they have also been applied to remove persistent organic pollutants [36] and inorganic pollutants, such as fluoride [37], from water. However, to the best of our knowledge, the removal of ARS dye molecules by adsorption onto magnetic chitosan core–shell network is yet to be investigated. 

Therefore, in the current study, we developed a magnetic chitosan core–shell network (MCN) and examined its adsorption potential towards the removal of ARS dye in aqueous solution under different conditions. In addition, to explain the adsorption mechanism and kinetics, the adsorption data were fitted to different isotherm and kinetic models. Furthermore, the desorption of ARS dye molecules from the exhausted MCN was approached by both chemical and thermal treatment. Overall, the obtained results revealed that the MCN could potentially be used as an adsorbent for the effective removal of ARS dye with subsequent reusability.

## 2. Materials and Methods

### 2.1. Materials

Medium molecular weight (190–310 kDa) chitosan powder and glutaraldehyde (25% solution in water) were obtained from Sigma-Aldrich. Glacial acetic acid 99.8–100.5%, hydrochloric acid (HCl), sodium hydroxide (NaOH) pellets, calcium chloride dihydrate (CaCl_2_·2H_2_O), and sodium chloride (NaCl) were purchased from VWR Chemicals BDH. Ferric chloride hexahydrate (FeCl_3_·6H_2_O) and ferrous sulfate heptahydrate (FeSO_4_·7H_2_O) were procured from Riedel-de Haën and Merck, respectively. Sodium bicarbonate (NaHCO_3_) and ARS (C_14_H_7_NaO_7_S) were bought from Carl Roth. Magnesium sulphate heptahydrate (MgSO_4_·7H_2_O) and indigo carmine (C_16_H_8_N_2_Na_2_O_8_S_2_) were attained from KMF Laborchemie Handels GmbH and Alfa Aesar, respectively.

### 2.2. Synthesis of Magnetic Chitosan Core–Shell Network (MCN)

**Scheme 1:**
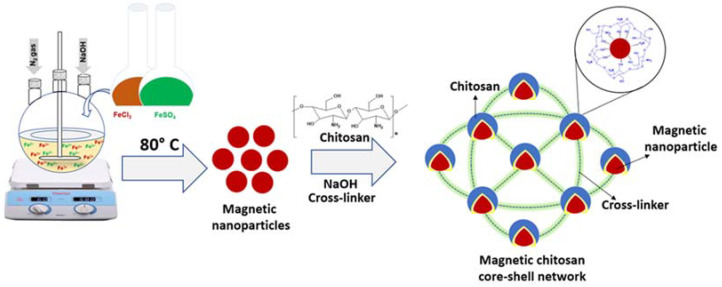
Schematic illustration of the MCN synthesis.

The MCN was obtained by a two-step process. Step 1-Synthesis of magnetic (Fe_3_O_4_) nanoparticles: FeCl_3_·6H_2_O and FeSO_4_·7H_2_O salts were dissolved in 250 mL of reverse osmosis (RO) (Osmose 190, Dennerle, Germany; <0.2 mg/L dissolved organic carbon; conductivity < 20 µS/cm) quality water such that the molar ratio of Fe^3+^ to Fe^2+^ was 2 to 1. This salt mixture was then added to a three-necked round-bottom flask under nitrogen atmosphere, heated to 70 °C, and stirred constantly at 300 revolutions per minute (rpm). After 20 min, 2M NaOH (100 mL) solution was added dropwise, and the resulting black precipitate was stirred constantly for 30 min and cooled down to room temperature, filtered, and washed with RO water several times until neutral pH was reached. Eventually, the Fe_3_O_4_ nanoparticles were dried in a hot air oven at 110 °C for 5 h and used in step 2. Step 2-MCN preparation: Co-precipitation method was chosen for preparing the MCN, as shown in Scheme 1. In this method, 2 g chitosan was first dissolved in 3% acetic acid solution. Then, 2 g Fe_3_O_4_ nanoparticles were dispersed in the chitosan solution and stirred continually at 300 rpm at room temperature. After 1 h, 1M NaOH was added dropwise to this mixture, yielding the magnetic chitosan precipitate. To this precipitate, 3% glutaraldehyde was added, and stirring was continued overnight. The resulting MCN was washed with RO water several times and then dried for 14 h in a hot-air oven at 110 °C. Finally, the dried MCN was milled using a planetary ball mill (Fritsch, PULVERISETTE 7) and used for the characterization and adsorption experiments. 

### 2.3. Characterization

MCN was characterized using environmental scanning electron microscopy (ESEM) coupled with energy dispersive X-ray analyzer (EDS) (Quanta 400 FEG, FEI, Munich, Germany). Samples were coated with gold before the SEM–EDS analysis. Attenuated total reflectance-Fourier transform infrared spectroscopy (ATR-FTIR) (Bruker, ALPHA-Platinum, Ettlinglen, Germany) was used to identify the functional groups present in the chitosan, Fe_3_O_4_ nanoparticles, and MCN. pH zero-point charge (pH_zpc_) was determined using solid addition method, reported elsewhere [38]. The pH of the solution was measured using pH electrode and different solution pH was realized by adding 100 mM NaOH/HCl.

### 2.4. Batch Adsorption Studies

Adsorption of ARS dye onto MCN was studied in synthetic model water (SMW, pH 6.8 ± 0.1), composed of 10 mM ARS dye, 30 mM CaCl_2_, 50 mM NaHCO_3_, and 20 mM MgSO_4_. Batch experiments were performed by varying contact time (0–120 min), MCN dosage (0–50 mg), and pH conditions (pH 3–10). All the adsorption experiments were carried out in duplicate at room temperature and the average was reported. In a typical adsorption process, a known amount of MCN was added to a glass flask containing 50 mL SMW solution and shaken with a mechanical shaker (Laboshake, Gerhardt, Germany) at 150 rpm. After a period of time, the MCN was magnetically separated, and the solution was filtered through a 0.45 µm cellulose acetate membrane (Ahlstrom GmbH, Germany) to obtain a dust-free filtrate. The filtrate was then analyzed for residual ARS dye at the maximum adsorption wavelength of 260 nm using a UV-Vis spectrophotometer (PerkinElmer, Lambda 20, Germany). The adsorption capacity and the removal efficiency were determined according to the following equations:

(1) $$q\;\left(\mathrm{mg}/g)={\;V}_{L}/m_{A}*(C_{0}-C_{e}\right)$$

(2) $$\mathrm{Removal}\;\left(\%\right)=\left(C_{0}-C_{e}\right)/C_{0}*100$$

where q is the adsorption capacity (mg/g); C_0_ and C_e_ are the initial and equilibrium ARS dye concentrations (mg/L); V_L_ is the volume of the ARS dye solution (L); and m_A_ is the mass of the adsorbent (g).

To identify the suitable adsorption isotherm and kinetic model, adsorption experiments were performed with different initial ARS dye concentrations (10, 20, 40, 80, and 100 mg/L) and with different contact times (0–60 min) at room temperature. The adsorption data obtained were fitted to the Langmuir [39] and Freundlich isotherm [40] models. The non-linear forms of the isotherm models are presented below.

Langmuir non-linear isotherm:

(3) $$q_{e}=q_{m}K_{L}C_{e}/\left(1+K_{L}C_{e}\right)$$

where $q_{m}$ is the maximum adsorption capacity (mg/g), K_L_ is the Langmuir isotherm constant (L/mg), $q_{e}$ is the equilibrium adsorption capacity (mg/g), and $C_{e}$ is the equilibrium concentration (mg/L). 

Freundlich non-linear isotherm:

(4) $$q_{e}=K_{F}C_{e}^{\left(\frac{1}{n}\right)}\;\;$$

where n is the adsorption intensity and K_F_ is the Freundlich isotherm constant (mg/g(L/mg)^1/n^).

Furthermore, the adsorption data were fitted to the following non-linear Lagergren pseudo-first order [41] and pseudo-second order [42] kinetic models.

Pseudo-first order:

(5) $$q_{t}={\;q}_{e}(1-\exp^{\left(-K_{1}t\right)})$$

where $q_{t}$ is the adsorption capacity (mg/g) at time ‘t’, K_1_ is the pseudo-first order rate constant (1/min), and t is time (min).

Pseudo-second order:

(6) $$q_{t}=q_{e\;}^{2}K_{2}t/\left(1+q_{e}K_{2}t\right)$$

where K_2_ is the pseudo-second order rate constant (1/min).

To study the influence of dissolved organic matter (DOM) on ARS dye adsorption onto MCN, flower soil extract that has been used in previous works [43] was chosen as a model surrogate for DOM. A series of stock solutions with different DOC (dissolved organic carbon) content was prepared in SMW, treated using MCN, and filtered through a 0.45 µm cellulose acetate membrane. Then, the filtrate was analyzed to determine the ARS dye removal by UV-Vis spectrophotometry (λ = 260 nm), DOC (dissolved organic content), and UV_254_. DOC measurements were performed using a total organic carbon analyzer (Shimadzu, TOC-L).

From the DOC and UV_254_ values, specific ultraviolet absorbance (SUVA) was also determined using the following equation.

SUVA (L/(mg∙m)) = UV_254_ (cm^−1^)/(DOC (mg/g)) × 100 cm/m (7)

SUVA analysis can be used to measure the amount of hydrophilic and hydrophobic compounds in the water. If the SUVA value of the filtrate is ≥4 L/(mg·m), this indicates the presence of hydrophobic, aromatic, and high molecular weight DOM fractions, if it is ≤3 L/(mg·m), this indicates the presence of non-humic, hydrophilic, and low molecular weight DOM fractions [44].

### 2.5. Desorption and Reuse Experiment

Experiments to desorb the ARS dye from the spent MCN were performed using a batch method. Due to the electrostatic repulsion forces, alkaline medium has been found to be very effective in desorbing the anionic dyes from the adsorbent surface [34,45]. In this study, 0.1 M NaOH was therefore chosen as an eluent to desorb the ARS dye from the spent MCN [34]. After adsorption equilibrium was achieved, MCN was magnetically separated, treated with 0.1 M NaOH solution for 60 min, washed with RO water several times until pH 7 was reached, and dried subsequently in a hot-air oven at 110 °C for 3 h. The dried MCN was then ready for the use in the next cycle of adsorption and desorption experiments as described above. This procedure was carried out five times; ARS dye removal was determined after each cycle.

### 2.6. Thermal Treatment of the Spent MCN

After the fifth reuse cycle, the ARS dye-loaded MCN was air-dried, placed in a boat-shaped crucible, and heated from 22 °C to 700 °C in a rotary kiln furnace with a ramping rate of 6.3 °C/min under a nitrogen atmosphere. Subsequently, the obtained N-doped magnetic carbon material was used as an adsorbent for the removal of various dyes in aqueous solutions.

## 3. Results and Discussion

### 3.1. MCN Characterization

**Figure 1:**
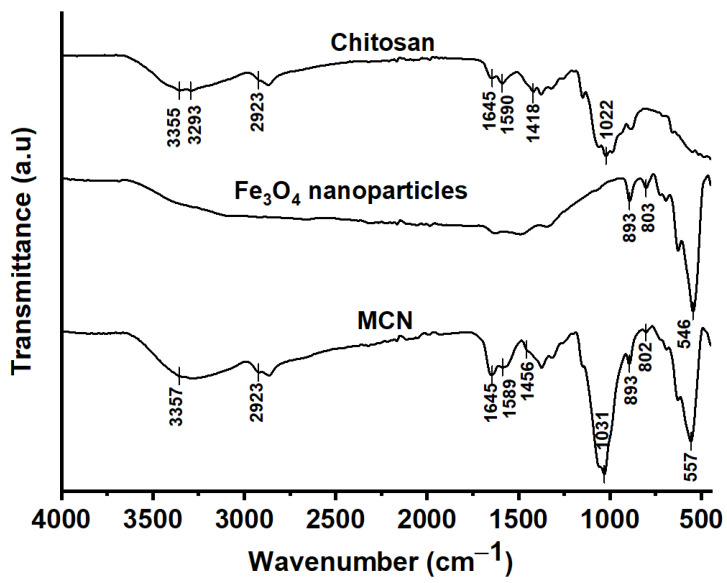
ATR-FTIR spectra of chitosan, Fe_3_O_4_ nanoparticles, and MCN.

Figure 1 shows the ATR-FTIR spectra of chitosan, Fe_3_O_4_, and MCN. Chitosan results in characteristic peaks at 1022, 1418, 1590, 1645, 2923, 3355, and 3293 cm^−1^ that corresponded to the stretching vibrations of C-O-C, C-N, C=O, C-H, N-H, and O-H, respectively. Pure Fe_3_O_4_ nanoparticles result in characteristic peaks at 546, 803, and 893 cm^−1^ that were assigned to Fe-O stretching mode, δ-OH, and γ-OH stretching vibrations, respectively, which confirm the presence of the most thermodynamically stable iron oxide (goethite) [46]. The reflectance of the characteristic peaks corresponding to both chitosan and Fe_3_O_4_ nanoparticles is also seen in the FTIR spectrum of MCN, but with a slight peak shift. In particular, the peak corresponding to the stretching vibration of Fe-O is shifted from 546 cm^−1^ to 557 cm^−1^ and the peak corresponding to the stretching vibration of N-H is shifted from 1418 cm^−1^ to 1456 cm^−1^, demonstrating the significant interactions (e.g., metal coordination and hydrogen bonding) between the Fe_3_O_4_ nanoparticles and chitosan during MCN preparation.

**Figure 2:**
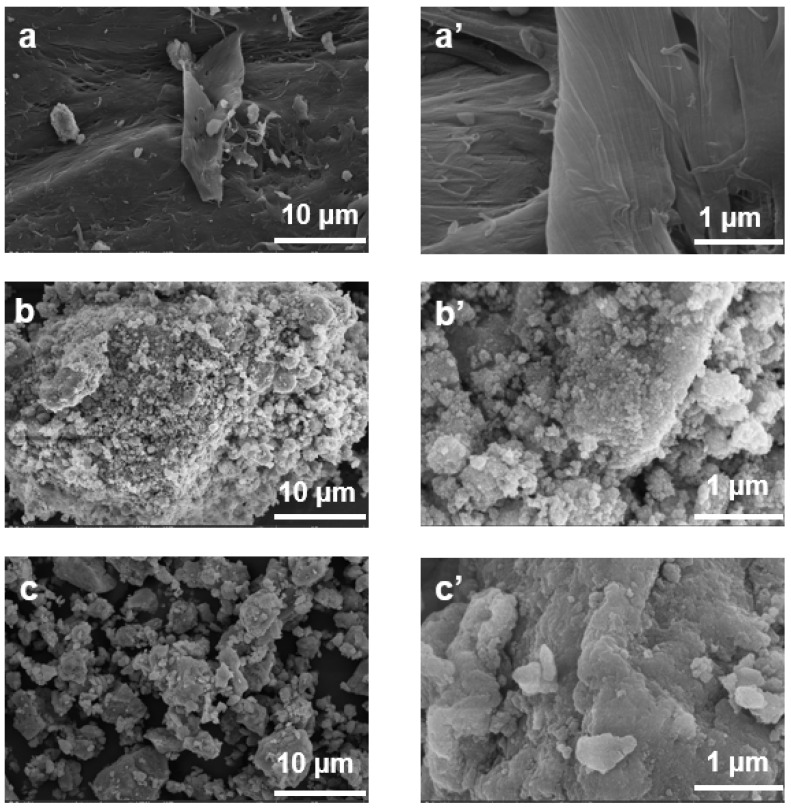
ESEM micrographs of (**a**,**a’**) chitosan, (**b**,**b’**) Fe_3_O_4_ nanoparticles, and (**c**,**c’**) MCN at lower (10 μm) and higher (1 μm) magnification.

It is obvious from Figure 2a,a’,b,b’ that the surface morphologies of raw chitosan and Fe_3_O_4_ nanoparticles are quite different from each other. Chitosan powder has a rough, flat, and smooth film-like surface structure [47], while Fe_3_O_4_ nanoparticles are spherical in shape [48]. It is evident from Figure 2c,c’ that after encapsulating Fe_3_O_4_ nanoparticles into chitosan matrix, the spherical shape of the Fe_3_O_4_ nanoparticles is diminished and nearly a core–shell network is formed [49]. The EDS spectrum of the MCN (see Figure S1, Supporting Information) shows distinctive peaks at 0.28, 0.39, 0.52, and 0.75 keV that correspond to C, N, O, and Fe, respectively, confirming the successful formation of MCN.

### 3.2. Removal of ARS Dye by Adsorption onto MCN

**Figure 3:**
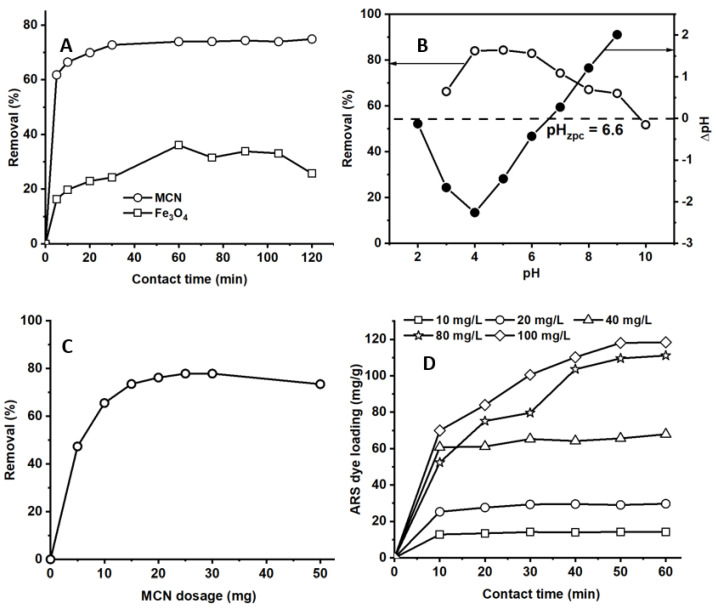
(**A**) Adsorption of the ARS dye onto MCN and Fe_3_O_4_ nanoparticles (experimental conditions: solution pH, 6.8; ARS dye concentration, 10 mg/L; MCN dosage, 25 mg; contact time, 0–120 min); (**B**) Influence of solution pH on ARS dye removal (experimental conditions: solution pH, 3–10; contact time, 60 min; ARS dye concentration, 10 mg/L, MCN dosage, 25 mg); (**C**) Influence of MCN dosage on ARS dye removal (experimental conditions: MCN dosage, 0–30 mg; solution pH, 6.8; ARS dye concentration, 10 mg/L; contact time, 60 min); (**D**) Effect of contact time and initial ARS dye concentration on ARS dye loading (experimental conditions: solution pH, 6.8; ARS dye concentration, 10–100 mg/L; MCN dosage, 25 mg; contact time, 0–60 min).

The removal of ARS dye by MCN and Fe_3_O_4_ nanoparticles was carried out as a function of contact time and the results are compared in Figure 3A. It is apparent that the MCN could rapidly achieve 62% ARS dye removal within the first five minutes, whereas Fe_3_O_4_ nanoparticles resulted in only 16% ARS dye removal. This might be due to the availability of more adsorption sites for the ARS dye at the beginning of the adsorption process. However, the ARS dye removal by MCN was not increased greatly after 60 min. Therefore, 60 min was fixed as a contact time for the subsequent experiments. In the case of Fe_3_O_4_ nanoparticles, the ARS dye removal increased with an increase in contact time up to 60 min and then decreased with a further increase in contact time, which might have been due to the occurrence of desorption of the ARS dye molecules with longer contact times. Similar results have already been reported for different dyes [50]. Compared to Fe_3_O_4_ nanoparticles, the MCN shows excellent performance for ARS dye removal due to the presence of additional functional groups in the MCN.

The pH of the dye solution influences the adsorption process by changing the zeta potential of the adsorbent [51]. Thus, ARS dye removal by MCN was examined when changing the pH of the SMW over the range from pH 3 to 10; the obtained results are shown in Figure 3B. It is obvious that the removal of ARS dye by the MCN is significantly affected by the solution pH and the removal is increased from 52% (q = 10.4 mg/g) to 84% (q = 17 mg/g) with decreasing pH, from pH 10 to pH 4. The removal of ARS dye remains almost the same (83–84%) between pH 4 and pH 6. With a further decrease in pH from pH 4 to pH 3, the ARS dye removal decreased. It is believed that under strong acidic conditions, the sulfonate groups (-SO_3_^−^) from the ARS dye combine with H^+^ ions and form -SO_3_H, resulting in a lower adsorption performance. A similar trend was also observed by Fan et al. [34]. Moreover, the pH_zpc_ of the MCN was determined as 6.6, suggesting that the The MCN surface was positively charged below pH 6.6 and negatively charged above pH 6.6. The ARS dye is negatively charged in aqueous solution; hence, the ARS dye molecules are attracted electrostatically below pH 6.6 and repelled electrostatically above pH 6.6. Further, in alkaline pH conditions, the ARS dye molecules may compete with the OH^−^ ions for the same adsorption sites, resulting in lower ARS dye removal. Nevertheless, the MCN retains 50% ARS dye removal efficiency even at pH 10, which can be attributed to the involvement of other driving forces, such as Van der Waals interactions and hydrogen bonding [5].

**Figure 4:**
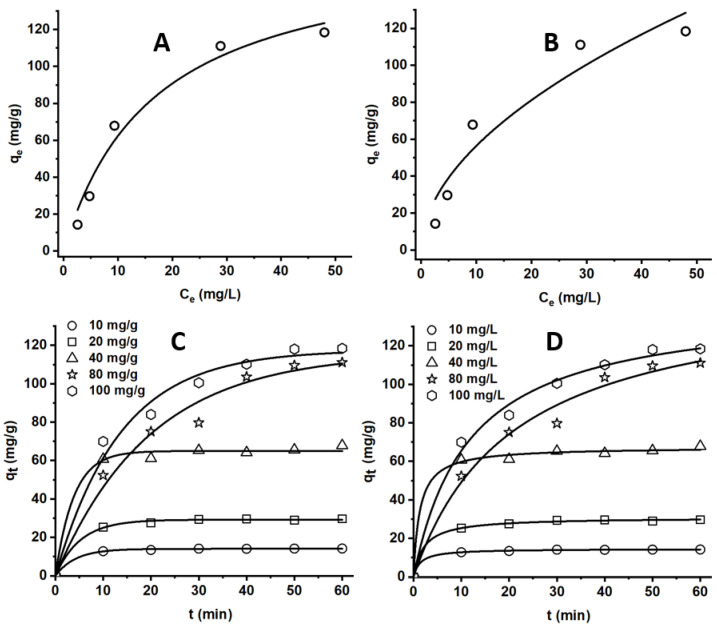
Non-linear (**A**) Langmuir and (**B**) Freundlich isotherm fitting curves; (**C**) Lagergren pseudo-first order and (**D**) pseudo-second order kinetic fitting curves obtained for the ARS dye adsorption onto the MCN.

As shown in Figure 3C, the ARS dye removal increased from 47% to 78% with an increase in MCN dosage from 0.005 g/L to 0.025 g/L, but then remained more or less constant up to the highest dosage of 0.05 g/L. Even a small reduction in the ARS dye removal from 78% to 74% could be observed. This might be due to the increase in number of adsorption sites available for the ARS dye molecules at increasing dosages, which at the same time led to the formation of agglomerates at higher dosages, decreasing the surface area available for the ARS dye [52]. Thus, the two effects oppose each other, resulting in optimal removal at a medium dosage. The removal of the ARS dye was examined as a function of the initial ARS dye concentration (10, 20, 40, 80, and 100 mg/L) and varied contact time (0–60 min); the obtained results are shown in Figure 3D. As expected, for the higher initial ARS dye concentrations and longer contact times, higher ARS loadings were achieved due to the greater adsorption of ARS dye molecules onto the MCN. The adsorption data obtained from this experiment were successfully fitted to the Langmuir and Freundlich non-linear isotherm models (see Figure 4A,B) and Lagergren pseudo-first order and pseudo-second order non-linear kinetic models (see Figure 4C,D). 

The determined values for the isotherm parameters are given in Table 1. Based on the correlation coefficient (R^2^) values shown in Table 1, the experimental data fits better to the Langmuir isotherm model than to the Freundlich isotherm model. This implied that the adsorption layer of the ARS dye onto the MCN surface occurred in a homogeneous monolayer. The determined Langmuir maximum adsorption capacity of MCN for the ARS dye was 166.4 mg/g. 

To assess the performance, the maximum adsorption capacity of MCN was compared with other adsorbents for the ARS dye removal, as presented in Table 2. In addition to facile synthesis, eco-friendly properties, and easy separation, it is evident from the comparison that the prepared MCN is superior to many of the adsorbents listed in Table 2.

**Table 2:** Comparison of maximum adsorption capacity (q_max_) for ARS dye removal with other adsorbents.

Adsorbent	q_max_	Reference
Poly(catechol–tetraethylenepentamine–cyanuric chloride)@hydrocellulose	284.1 mg/g	[53]
Activated carbon engrafted with Ag nanoparticles	232.6 mg/g	[54]
Gold nanoparticles loaded on activated carbon	123.4 mg/g	[55]
Activated carbon	85.2 mg/g	[56]
Molecularly imprinted magnetic chitosan	40.1 mg/g	[34]
Polyethyleneimine (PEI)-functionalized magnetic carbon nanotubes	196.1 mg/g	[57]
Polypyrrole-coated magnetic nanoparticles	116.3 mg/g	[58]
Activated carbon/γ-Fe_2_O_3_ nano-composite	108.7 mg/g	[59]
NiFe_2_O_4_/polyaniline magnetic composite	186 mg/g	[60]
Magnetic chitosan core–shell network	166.4 mg/g	This study

Table 3 provides the obtained values for the different kinetic parameters. It is apparent from the R^2^ value that the pseudo-second order kinetic model fits better to the adsorption data than to the Lagergren pseudo-first order (see Table 2) model, confirming that the rate of adsorption of the ARS dye onto the MCN can be considered as a chemisorption process [34].

### 3.3. Influence of DOM (as DOC) Concentration on ARS Dye Removal

**Figure 5:**
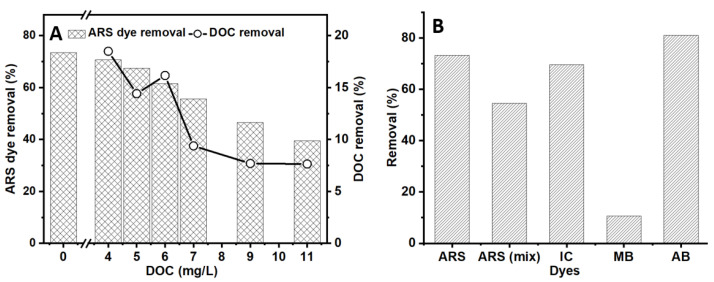
(**A**) Influence of DOM (as DOC) concentration on the removal of ARS dye and DOC by the MCN (experimental conditions: DOC concentration, 4–11 mg/L; solution pH, 6.8; ARS dye concentration, 10 mg/L; MCN dosage, 25 mg; contact time, 60 min); (**B**) Removal of different dyes using MCN (experimental conditions: solution pH, 6.8; dye concentration, 10 mg/L; MCN dosage, 25 mg; contact time, 60 min).

Wastewaters contain not only the ARS dye, but also DOM, which results in unavoidable interference during the adsorption process. Therefore, it is important to investigate the ARS dye adsorption onto the MCN also in the presence of DOM. It can be seen from the results (see Figure 5A) that the presence of DOM in SMW has a negative impact on the ARS dye removal. After 60 min of contact time, the ARS dye removal decreased by 45% (from 73% to 40%) with an increase in DOC concentration from practically 0 to 11 mg/L, revealing the significant influence of DOM on ARS dye removal. The same effect was reported in another context by Guillossou et al., who studied the competition between DOM and 12 organic micropollutants (OMPs) during adsorption onto powdered activated carbon dosed in either ultra-pure water or wastewater effluent (DOC 7.3 mg/L) for contact times of 30 min and 72 h [61]. They found that the OMP removal after a contact time of 30 min decreased on average by 73% and by 30% after 72 h and concluded that the lower removal could be attributed to competition for the same adsorption sites between DOM and OMPs and a hinderance for OMP diffusion due to pore blockage. In addition, the electrostatic repulsive forces between the previously adsorbed DOM onto MCN and ARS dye (i.e., both are negatively charged) might result in lower ARS dye removal [61].

This was further confirmed by comparing DOC and SUVA values before and after treatment. The DOC removal ranged from 19% to 8% for DOC background concentrations from 4 to 11 mg/L, demonstrating the occupation of adsorption sites by DOM compounds. The SUVA values determined for the SMW with different DOM content before and after treatment are shown in Figure S2 and ranged between 16.7 and 10.3 L/(mg·m) and from 6.2 to 7.1 L/(mg·m), indicating (≥4 L/(mg·m)) the presence of high content of hydrophobic, aromatic, and high molecular weight DOM fractions [44]. Notably, SUVA values obtained for the treated water had lower hydrophobic, aromatic, and high molecular weight DOM fractions than before treatment, confirming the preferential adsorption of hydrophobic, aromatic, and high molecular weight DOM fractions onto the MCN.

### 3.4. Adsorption of Various Dyes onto MCN

To investigate the capability of MCN to adsorb various kind of dyes, the adsorption of indigo carmine (IC), Alcian blue (AB), and methylene blue (MB) dyes onto the MCN was examined in a single system and the results are compared with those for ARS dye removal. According to the results shown in Figure 5B, the MCN had similar performance for the anionic dyes ARS (73%), IC (70%), and AB (81%), but showed poor performance for the cationic dye MB (11%). These results clearly exhibited that the developed MCN was better at removing anionic dyes than cationic dyes. On the other hand, the prepared MCN showed a distinct difference in ARS dye removal (55%) from the mixture of ARS+IC+AB+MB (ARS (mix)) dye solutions compared to 73% in a single system. This might be because of the competition between the different dyes for the same adsorption sites. 

### 3.5. Regeneration and Reuse of Spent MCN

**Figure 6:**
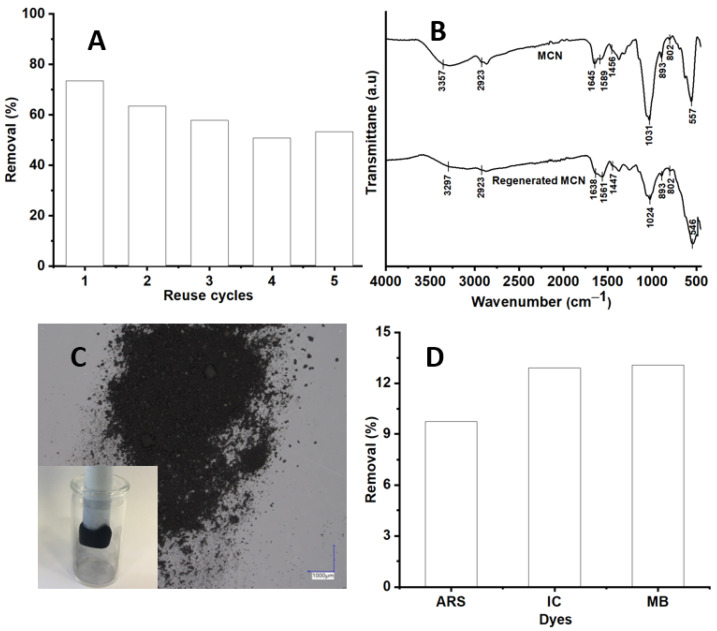
(**A**) Reuse cycle analysis of MCN for the removal of ARS dye; (**B**) ATR-FTIR spectra of MCN and regenerated MCN; (**C**) Optical image of produced N-doped magnetic carbon and magnetic properties of N-doped magnetic carbon; (**D**) Removal of ARS, IC, and MB dyes by the N-doped magnetic carbon (experimental conditions: solution pH, 6.8; dye concentration, 10 mg/L; N-doped carbon dosage, 25 mg; contact time, 60 min).

The sustainable reuse of an adsorbent is very important for its widespread applications. Therefore, the regeneration and reuse of the spent MCN were examined for five consecutive cycles and the results are shown in Figure 6A. The ARS dye removal efficiency decreased as the number of reuse cycles increased; however, the MCN retained almost 73% of its original efficiency at the end of the fifth cycle. ATR-FTIR analysis was therefore performed to identify either incomplete ARS desorption or a loss of stability of the MCN after five consecutive desorption and reuse processes (i.e., after the fifth cycle). In addition to noticeable changes in peak intensity, it was apparent from the ATR-FTIR spectra of the regenerated MCN (see Figure 6B) that there was a shift in peak position compared to those of the virgin MCN, suggesting the occurrence of intermolecular interactions during the regeneration process. At the same time, no peaks corresponding to the ARS dye molecule were observed, indicating complete desorption of the ARS dye molecules. The decrease in removal efficiency might thus be a result of the alteration of the MCN surface properties during the adsorption–desorption processes.

### 3.6. Thermal Treatment of Spent MCN

N-doped carbon materials have been produced successfully from chitosan via pyrolysis process and used in various applications [62,63]. The aim of this experiment was therefore to produce N-doped magnetic carbon material from the spent MCN. In this context, after the fifth reuse cycle, the ARS dye-loaded MCN was pyrolyzed under given experimental conditions to produce N-doped magnetic carbon (see Figure 6C) and subsequently used as the adsorbent for the removal of ARS, IC, and MB dyes in aqueous solution. It is obvious from the inserted image in Figure 6C that the magnetic property of the material was retained even after the pyrolysis process; hence it can be easily separated by an external magnetic field. Further, it can be seen from Figure 6D that the produced N-doped magnetic carbon was able to remove 10%, 13%, and 13% of ARS, IC, and MB dyes, respectively. Though the produced N-doped magnetic carbon is less efficient for dye removal than the MCN, the adsorption properties of N-doped magnetic carbon could be most likely enhanced by performing the pyrolysis under other experimental conditions. Further characterization studies are also needed to identify the physicochemical properties of the N-doped magnetic carbon.

## 4. Conclusions

In this work, a magnetic chitosan core–shell network (MCN) was successfully synthesized for the sustainable removal of alizarin red S (ARS) dye in aqueous solution. It was deduced from the batch experiments that the ARS dye adsorption onto the MCN was quite fast, increased with increasing contact time, and reached equilibrium within 60 min. Noticeable ARS dye removal occurred in acidic conditions due to electrostatic interactions. The adsorption process was well described by the Langmuir isotherm and followed pseudo-second order kinetics. The Langmuir maximum monolayer adsorption capacity of the MCN for the ARS dye was determined to be 166.4 mg/g, which was higher than for many other adsorbents. The prepared MCN showed almost similar adsorption behavior to other anionic dyes such as indigo carmine and Alcian blue. In the presence of a 11 mg/L dissolved organic content (DOC) and a contact time of 60 min, the removal of the ARS dye decreased from 73% to 40%. This may have been due to a combination of pore blockage, competition between the ARS dye, and dissolved organic matters (DOMs) for the same adsorption sites, or electrostatic repulsive forces between the ARS dye in solution and DOM adsorbed onto MCN, or these factors individually. Furthermore, regeneration and reuse experiments showed that more than 70% of the original removal efficiency was retained, even after five consecutive cycles. Most importantly, the exhausted MCN was pyrolytically converted into N-doped magnetic carbon and explored as an adsorbent for different dyes. Nevertheless, further studies are required to optimize the pyrolysis process and to improve the physicochemical properties of the N-doped magnetic carbon.

## Figures and Tables

**Table 1:** Langmuir and Freundlich isotherm parameters for ARS dye adsorption onto the MCN.

	Langmuir Isotherm			Freundlich Isotherm		
	q_max_ (mg/g)	K_L_ (L/mg)	R^2^	n	K_F_ (mg/g(L/mg)^1/n^)	R^2^
ARS dye	166.4	0.06	0.9637	1.9	16.9	0.8944

**Table 3:** Lagergren pseudo-first order and pseudo-second order kinetic parameters for the ARS dye adsorption onto the MCN.

ARS Dye Concentration C_0_ [mg/L]	q_e,exp_ (mg/g)	Pseudo-First Order			Pseudo-Second Order		
		K_1_ (1/min)	q_e,model_ (mg/g)	R^2^	K_2_ (1/min)	q_e,model_ (mg/g)	R^2^
10	14.2	0.24	14.1	0.9979	0.05	14.5	0.9994
20	29.7	0.19	29.2	0.9975	0.02	30.8	0.9988
40	67.9	0.26	65	0.9926	0.01	67.4	0.9962
80	111.1	0.05	116	0.9776	0.003	141.7	0.9835
100	118.4	0.07	118	0.9823	0.006	150.5	0.9927

## Data Availability

The data presented in this study are available on request from the corresponding author.

## Supplementary Materials

The following are available online at https://www.mdpi.com/article/10.3390/ma14247701/s1, Figure S1: EDS spectra of MCN, Figure S2: Comparison of SUVA values for waters with different DOC concentration (before and after treatment).
