# Supplementary material for: Sustainable Development of Magnetic Chitosan Core–Shell Network for the Removal of Organic Dyes from Aqueous Solutions
Source: Materials (Basel). 2021 Dec 13;14(24):7701. doi: 10.3390/ma14247701 (PMC8706649; doi:10.3390/ma14247701)
Supplement: Supplementary file 1 [file materials-14-07701-s001.zip › materials-1503021-supplementary.pdf]

materials

Supporting information

Sustainable development of magnetic chitosan core-shell network for the removal of organic dyes in aqueous solutions

Karthik Rathinam[1,*], Xinwei Kou[1], Ralph Hobby[1] and Stefan Panglisch[1,2,3,4,*]

[1] Chair for Mechanical Process Engineering / Water Technology, University of Duisburg-Essen, Duisburg, Germany
[2] IWW Water Centre, Mülheim an der Ruhr, Germany
[3] DGMT German Society for Membrane Technology e.V., Essen, Germany
[4] Centre for Water and Environmental Research (ZWU), Essen, Germany
* Correspondence: karthik.rathinam@uni-due.de (K.R); stefan.panglisch@uni-due.de (S.P)

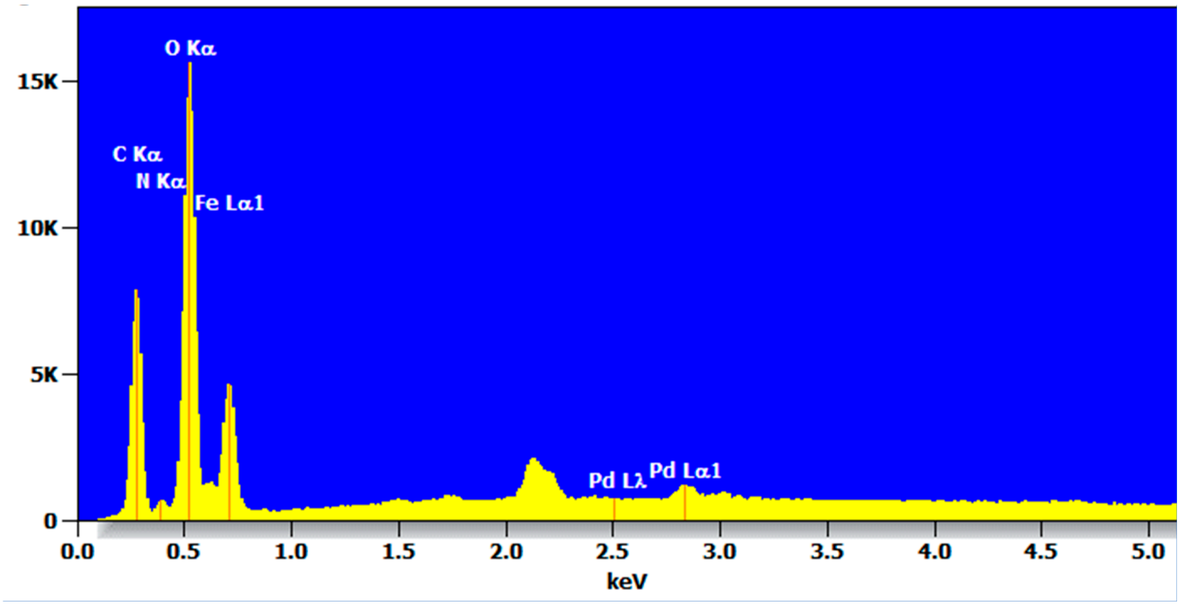

Figure S1. EDS spectra of MCN.

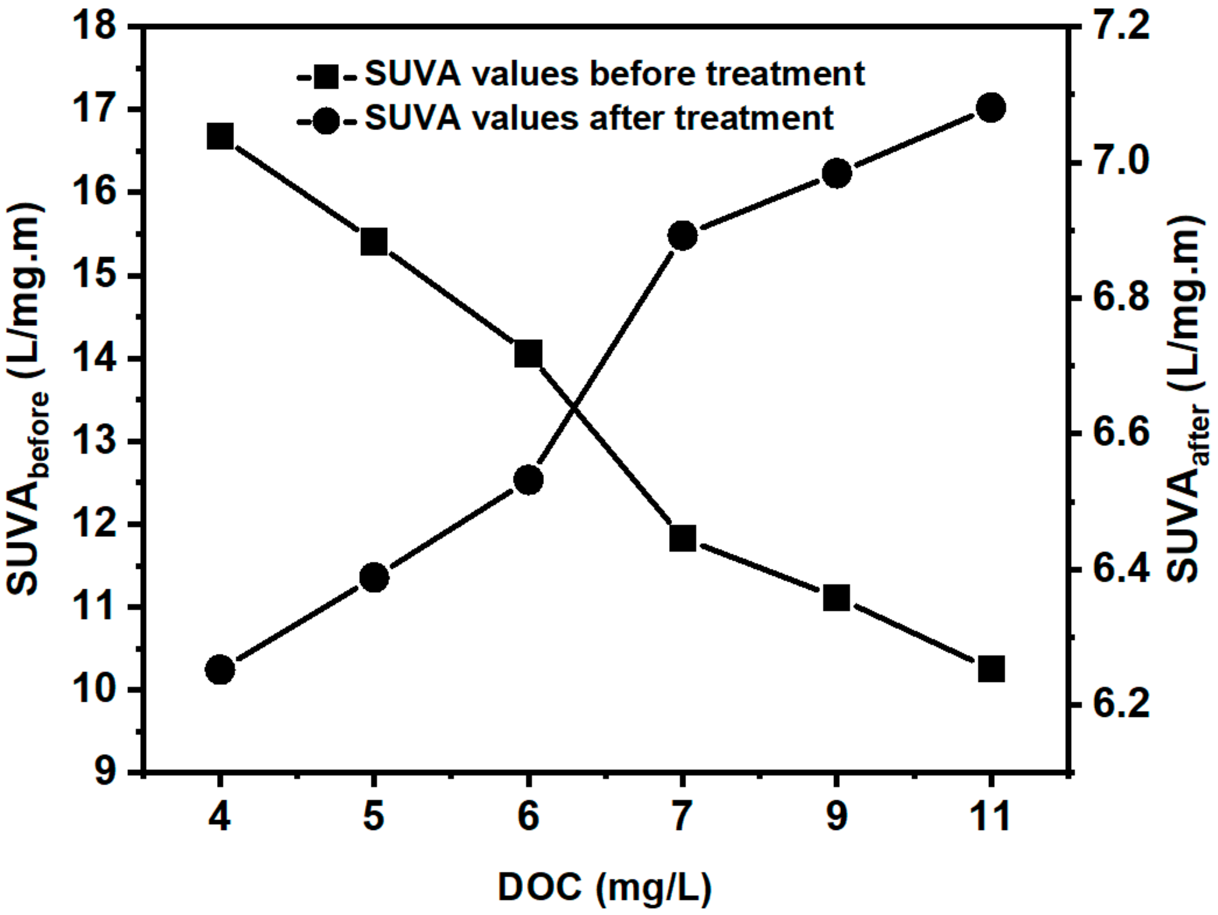

Figure S2. Comparison of SUVA values for waters with different DOC concentration (before and after treatment).
